# Regulatory Frameworks for AI-Enabled Medical Device Software in China: Comparative Analysis and Review of Implications for Global Manufacturer

**DOI:** 10.2196/46871

**Published:** 2024-07-29

**Authors:** Yu Han, Aaron Ceross, Jeroen Bergmann

**Affiliations:** 1 University of Oxford Oxford United Kingdom; 2 Department of Technology and Innovation The University of Southern Denmark Denmark Denmark

**Keywords:** NMPA, medical device software, device registration, registration pathway, artificial intelligence, machine learning, medical device, device development, China, regulations, medical software

## Abstract

The China State Council released the new generation artificial intelligence (AI) development plan, outlining China's ambitious aspiration to assume global leadership in AI by the year 2030. This initiative underscores the extensive applicability of AI across diverse domains, including manufacturing, law, and medicine. With China establishing itself as a major producer and consumer of medical devices, there has been a notable increase in software registrations. This study aims to study the proliferation of health care–related software development within China. This work presents an overview of the Chinese regulatory framework for medical device software. The analysis covers both software as a medical device and software in a medical device. A comparative approach is employed to examine the regulations governing medical devices with AI and machine learning in China, the United States, and Europe. The study highlights the significant proliferation of health care–related software development within China, which has led to an increased demand for comprehensive regulatory guidance, particularly for international manufacturers. The comparative analysis reveals distinct regulatory frameworks and requirements across the three regions. This paper provides a useful outline of the current state of regulations for medical software in China and identifies the regulatory challenges posed by the rapid advancements in AI and machine learning technologies. Understanding these challenges is crucial for international manufacturers and stakeholders aiming to navigate the complex regulatory landscape.

## Background

New software solutions that are being developed, especially medical devices that combine artificial intelligence (AI) and machine learning (ML), show a huge potential for patient benefit. These kinds of applications can be used across different medical conditions, with the potential for easy scale-up to larger populations. It can reduce the burden on health care professionals and decrease the possible risk of missing vital information. For example, radiology software is used to screen and diagnose large amounts of X-ray images [1]. A combined AI and ML approach can also be applied in, for example, oncology for the next‐generation sequencing [2], in ophthalmology for image recognition [3], or as a support system for general medical decision-making [4]. ML models have been used for anything from improving outcomes for diabetic patients [5] to tuberculosis diagnosis [6]. Many of these approaches should be applicable on a global scale, and thus there is a growing interest in applying these solutions across borders. This has led clinicians, academics, and manufacturers to look at China and its medical device regulatory environment. However, navigating China's regulatory environment presents inherent complexities stemming from language barriers, geographical distances, and a general lack of familiarity with the regulatory framework. These complexities are augmented by innovative products that can have unconventional regulatory requirements. Easing these barriers holds the potential to facilitate the seamless exchange of solutions across international boundaries, fostering mutual opportunities. This paper provides a regulatory view of China, the biggest booming market for medical device software, and discusses the implications for global manufacturers.

## China AI Development Plan

The 21st century has seen a rapid development of the Chinese economy and its ability to produce, manufacture, and distribute technology. In 2017, the China State Council published a white paper discussing a *new generation AI development plan* [7]. The document indicated that the number of AI scientific papers published and invention patents granted in China ranked second worldwide. Several domain-specific applications that were developed in China have gained widespread attention, including intelligent monitoring, biometric recognition, industrial robots, service robots, and unmanned driving. The AI Development Plan clearly states China’s support for smart medical care, products, and services that use AI. Moreover, it is stated that this even should be developed as a priority. The vision is to establish a major medical system that leverages AI and ML.

China has become a major global producer and consumer of medical devices [8]. With one of the world’s largest populations (1.426 billion in 2022) [9], the need is obvious in terms of access to medical technology. In 2019, the Chinese medical device market had an estimated revenue value of 629 billion RMB (US $88.7 billion), more than double of what it was in 2015 (308 billion RMB or US $44.2 billion) before the plan was released [10]. This coincides with a growing trend of medical device software (MDSW) registrations [11]. One factor driving this trend is the potential that digital health offers in terms of ease of scalability, which provides an opportunity to advance health care more sustainably.

Global manufacturers seeking to enter the Chinese market must possess a profound comprehension of the regulatory landscape governing MDSW. This necessitates a thorough grasp of the intricacies surrounding registration prerequisites, regulatory oversight, disparities vis-à-vis regulatory bodies in alternative geographic regions, and the contemporary device taxonomy specific to China. Simultaneously, researchers and health care practitioners must remain vigilant by staying abreast of the latest developments transpiring within the Chinese milieu. The global pandemic has unequivocally underscored the imperative of comprehending and navigating policies and regulations in foreign jurisdictions, including but not limited to China, as an indispensable facet of effectively addressing worldwide crises. By extension, software-based solutions can similarly accrue significant advantages through adopting a holistic and globally informed perspective.

## Chinese Regulation on Medical Device Software

After *the new generation AI development plan* was introduced, China’s medical products regulatory authority—National Medical Products Administration (NMPA)—released many regulations to fit the plan’s theme. In 2022, the NMPA launched a program on digital health. Two MDSW guidelines were published as part of this program. Table 1 shows a series of regulatory documents published with regard to MDSW and AI-enabled software. The NMPA released the first document in 2015, while a more up-to-date document was made public in 2022. This updated version raised more detailed requirements for the whole life cycle management of these technologies, as well as for quality management, verification, raw code analysis, and safety management.

**Table 1:** China National Medical Products administration (NMPA) regulatory documents for medical device software.

Date of publication	Regulatory document
August 2015	Guidelines of medical device software registration and review [12]
July 2019	Key points of deep learning decision-making assisting medical device software review [13]
July 2021	Guidelines for the classification and designation of artificial intelligence medical software [14]
March 2022	Guidelines of medical device software registration and review [15]
August 2022	Guidelines for the classification and designation of artificial intelligence medical software [16]

Several standards are referenced in the regulation, and they include (but are not limited to) standards on the risk level of software (YY/T0664-2008), on software engineering (GB/*t* 19003-2008), and those that describe the medical device quality management requirements (YY/*t* 0287-2003). These standards can help with compliance with these new regulations, and this provides a useful function in the regulatory pathway.

In China, MDSW includes “software as a medical device” (SaMD) and “software in a medical device” (SiMD). The term “software as a medical device” is defined by the International Medical Device Regulators Forum (IMDRF) as software intended to be used for one or more medical purposes without being part of a hardware medical device [17]. This delineation posits the software itself as a standalone medical device. Conversely, “SiMD” denotes software that functions as an integral constituent of an entire medical apparatus, such as its involvement in the operation of magnetic resonance imaging scanners, x-ray machines, or insulin pumps. In these cases, the software and other components all fall under the same registration license “SiMD.” It is noteworthy that in China, software harnessing AI or ML technologies may concurrently straddle both the SaMD and SiMD categories.

**Figure 1:**
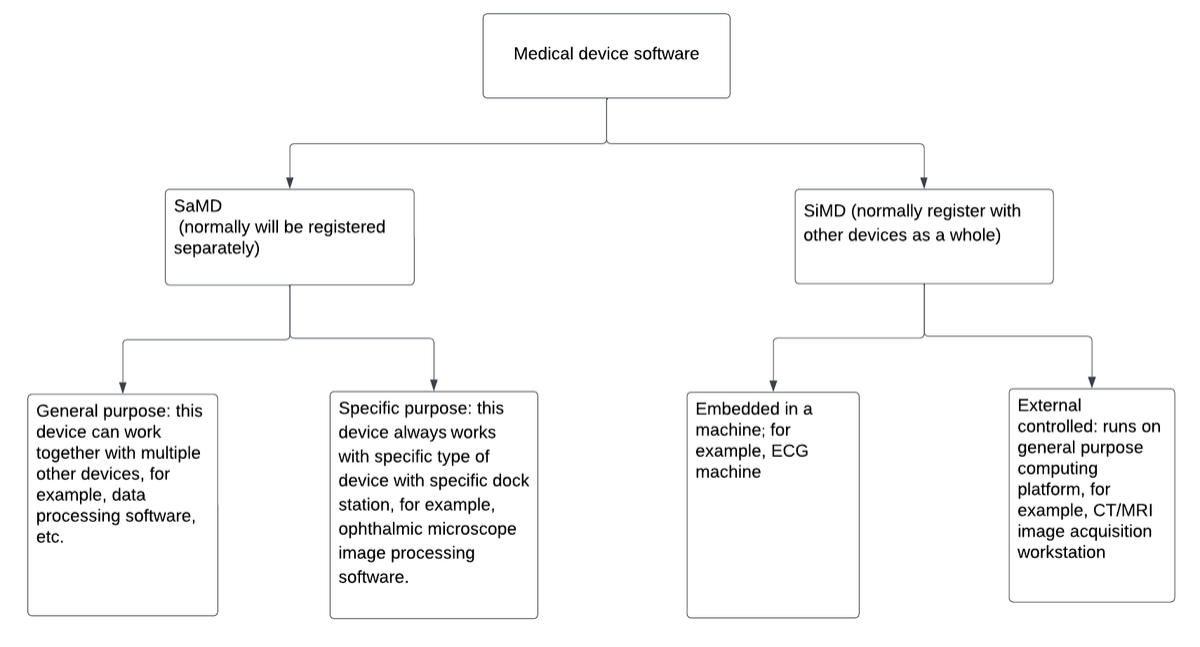
Categories of medical device software. CT: computed tomography; ECG: electrocardiography; MRI: magnetic resonance imaging; SaMD: software as a medical device; SiMD: software in a medical device.

An overview is given in Figure 1 with regard to how software devices are categorized from a function or a design perspective. Devices are initially split into SaMD and SiMD. SaMD is normally registered separately, while, as mentioned previously in the case of SiMD, the software is often registered along with other components [15]. In the case of SiMD, the software doesn’t have its own classification, but it shares the same classification with other parts of the device. The final classification would then be based on the risk of the whole device. SaMD can be split into 2 types depending on its purposes. Its purpose can be (1) general or (2) specific. For the general-purpose definition, the device can work together with multiple other devices, as happens in the example of data processing software. For the specific purpose case, the device always works with a distinct set of devices for a particular purpose. An illustration of this is the ophthalmic microscope image processing software. The SiMD also consists of 2 types of devices. One type is embedded in a machine (eg, an electrocardiogram machine), while the other type is externally controlled. A general-purpose computing platform (eg, a computed tomography [CT] and magnetic resonance image acquisition workstation) is a good exemplification of an externally controlled type of SiMD. The categorization of the software is a crucial step in the regulatory journey of a product.

## Regulatory Environment in China

The oversight and governance of medical devices within China are primarily administered by the Center for Medical Device Evaluation, an integral component of the NMPA. The regulatory landscape formulated by the NMPA to govern medical devices is predicated upon a comprehensive framework rooted in Chinese legislation, regulations, and advisory directives. This multifaceted regulatory apparatus encapsulates various facets pertinent to market entry, encompassing the specification of device categories, the classification of devices, the requisite content of registration review dossiers, and the imperative facet of post-market surveillance. In conformity with these regulatory imperatives, manufacturers need to engage proactively with the NMPA, necessitating their involvement across all aforementioned dimensions.

Medical devices are subject to regulatory oversight within a risk management framework that stratifies these products according to risk levels, ranging from low risk (class I) to high risk (class III). In the case of manufacturers engaging in the importation of medical devices into China, the responsibility for the review process falls under the purview of national authorities. Concurrently, certain domestically produced medical devices are subject to regulatory scrutiny by provincial authorities. The classification of a medical device within the Chinese regulatory context necessitates the alignment of its device description with the pertinent information contained within the medical device catalog [18].

In general, manufacturers possess 2 principal avenues for conceiving innovative medical equipment, which are occasionally amenable to synergistic integration. The first approach involves commencing with a patient-centered needs assessment (need-led innovation) to engender a “novel” technological solution. The alternative approach entails the development of a “novel” technology, with the subsequent identification of a correlating patient need [19]. These innovations can occur either before or after appropriate regulations have been set [20]. It is common that transformative ideas initially do not have suitable regulations in place and that this mismatch can lead to either delays in market adoption or concerns in terms of device performance and safety. However, any medical software enterprise aspiring to introduce its product to the market is mandated to adhere to prevailing regulatory mandates. Accordingly, a comprehensive comprehension of the product's classification and regulatory prerequisites within a specific market is of paramount significance, as the realms of innovation and regulation engage in a dynamic interplay. A good understanding is particularly important, as it has been suggested that the complexity of medical device regulations can increase whenever new regulations are formed [21]. Erroneous classification of product risk and the correlated regulatory obligations can result in exacerbated time and financial investments for subsequent rectification. Thus, the incorporation of regulatory considerations should be undertaken expeditiously, as many decisions regarding the final product are already made at the early stages of the research and development process.

## Specific Rules for Software and AI

**Figure 2:**
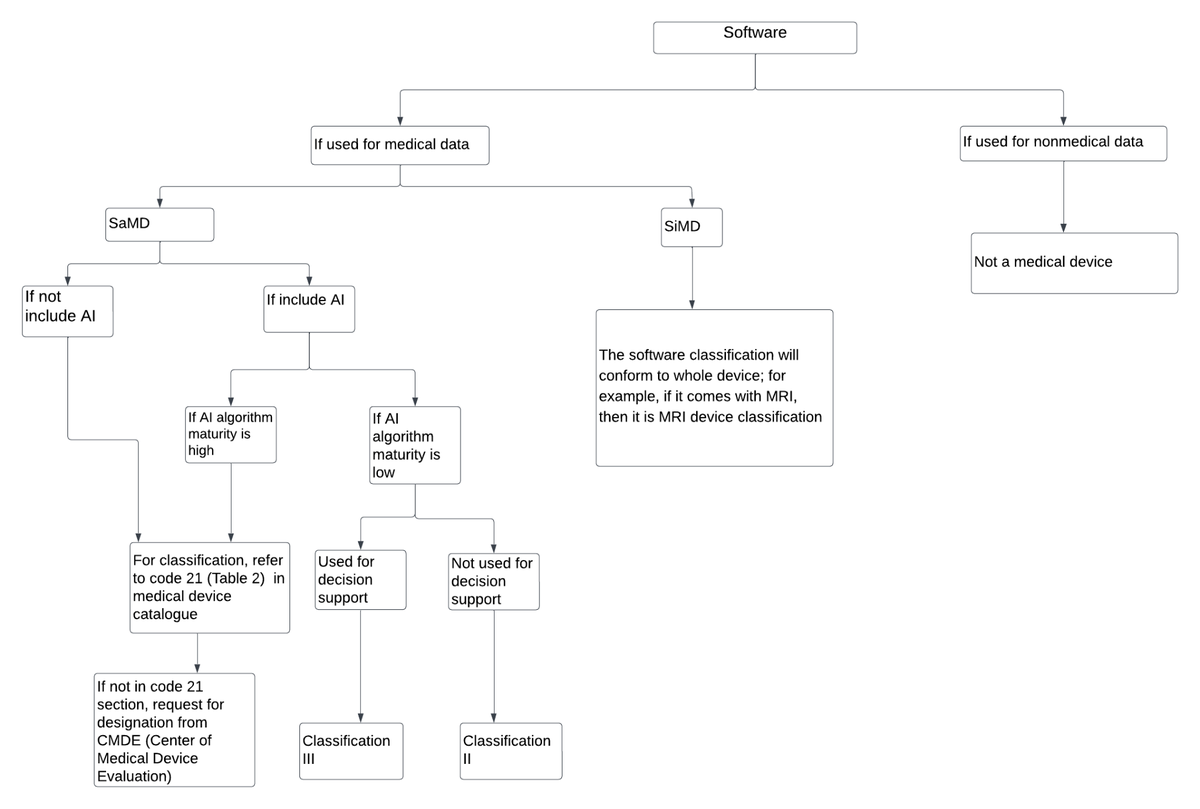
Medical device software classification flowchart. AI: artificial intelligence; MRI: magnetic resonance imaging; SaMD: software as a medical device; SiMD: software in a medical device.

Medical software is basically divided into auxiliary diagnosis and treatment devices according to their intended use. A detailed translation of the software catalog can be found in Multimedia Appendix 1. The SaMD (which begins with code 21 according to regulation) is categorized into 6 categories: treatment planning software (21-01), image processing software (21-02), data processing software (21-03), decision support software (21-04), in vitro diagnostic software (21-05), and other software (21-06). If the device to be registered is not included in the list, then it has to be re-classified through the device designation pathway [22]. A simple flowchart for the classification of the software is shown in Figure 2.

There are 2 branches for SaMD, which are split between AI and other technologies. If AI is applied, then a further decision is made according to the level of maturity of the algorithm. A high maturity level of the algorithm signifies that the safety and efficacy profiles of the algorithm have been judiciously established, while conversely, a lower degree of maturity implies that such establishment has not been ascertained. A preconsultation meeting could be used to discuss the maturity level with the NMPA. If the AI algorithm has a well-established profile, then manufacturers can refer back to Multimedia Appendix 1, code 21 [18] for classification. A request for designation could then be sent to the NMPA, if the device is out of scope. If the maturity degree is low, then there are 2 classifications possible. The device could be classified as a class III device if it is used for decision support; otherwise, the device will be assigned a classification of II, which represents a lower risk class. According to the Medical Device Classification Catalog [18], a class II device classification is given when the software does not contain any AI and the medical software is only used for image and data processing, thus not used for diagnostics. If it were used for diagnostic purposes, then the classification would become III. The degree of risk for diagnostic software is determined by the level of maturity, registration of the applied algorithm in their database, and the “object” of interest (referring to a particular disease, such as a certain type of cancer) [23].

However, if the software just provides diagnostic suggestions through its algorithm (in other words, it only has an auxiliary diagnostic function and does not directly provide a diagnostic conclusion), then the device can be regulated as a class II medical device. Yet, if the diagnostic software automatically recognizes, for instance, a lesion site through its algorithm and provides clear diagnostic prompts, a class III classification would be assigned due to the increased relative risk. In general, medical software using AI technology is currently managed by designating it the highest possible classification in China. This is driven by the novelty of the technology, as well as the lack of in-depth and complete evaluations of the clinical risks. China has been focusing more on reviewing the algorithm itself, while in the United States, attention has shifted toward the manufacturers themselves [24].

It should be noted that not all software applied in the medical field is regulated as a medical device by the NMPA. If the software is used to process medical device data for measurement, model calculation, or analysis, then it is deemed MDSW and thus regulated by NMPA. If the software is used for non-medical device data, it will not be regulated as a medical device under the NMPA. This is the case when software is used for the processing of general patient information or for patient testing reports, both of which are not seen as medical device data.

China’s and the IMDRF criteria share many similarities on how to determine if the software is a SaMD. According to the IMDRF [25], the SaMD definition should include a clear statement about the intended use of the device, and the following aspects need to be described in order to be able to be regulated as SaMD (Textbox 1).

In alignment with the IMDRF, the European Medicines Agency declares that only devices whose intended use includes a medical purpose and influences the patient’s health care situation can be deemed to be medical devices. Products such as medical information management software (which is a hospital management tool) are also not designated as medical devices. This is similar to China, since it then does not meet the definition of a medical device.

**Aspects need to be described in order to be able to be regulated as software as a medical device (SaMD).**

- The “significance of the information provided by the SaMD to the health care decision,” which is used to identify the intended medical purpose of the SaMD.
- The “state of the health care situation or condition” that the SaMD is intended for.

## General Registration Process and Clinical Evaluation

**Figure 3:**
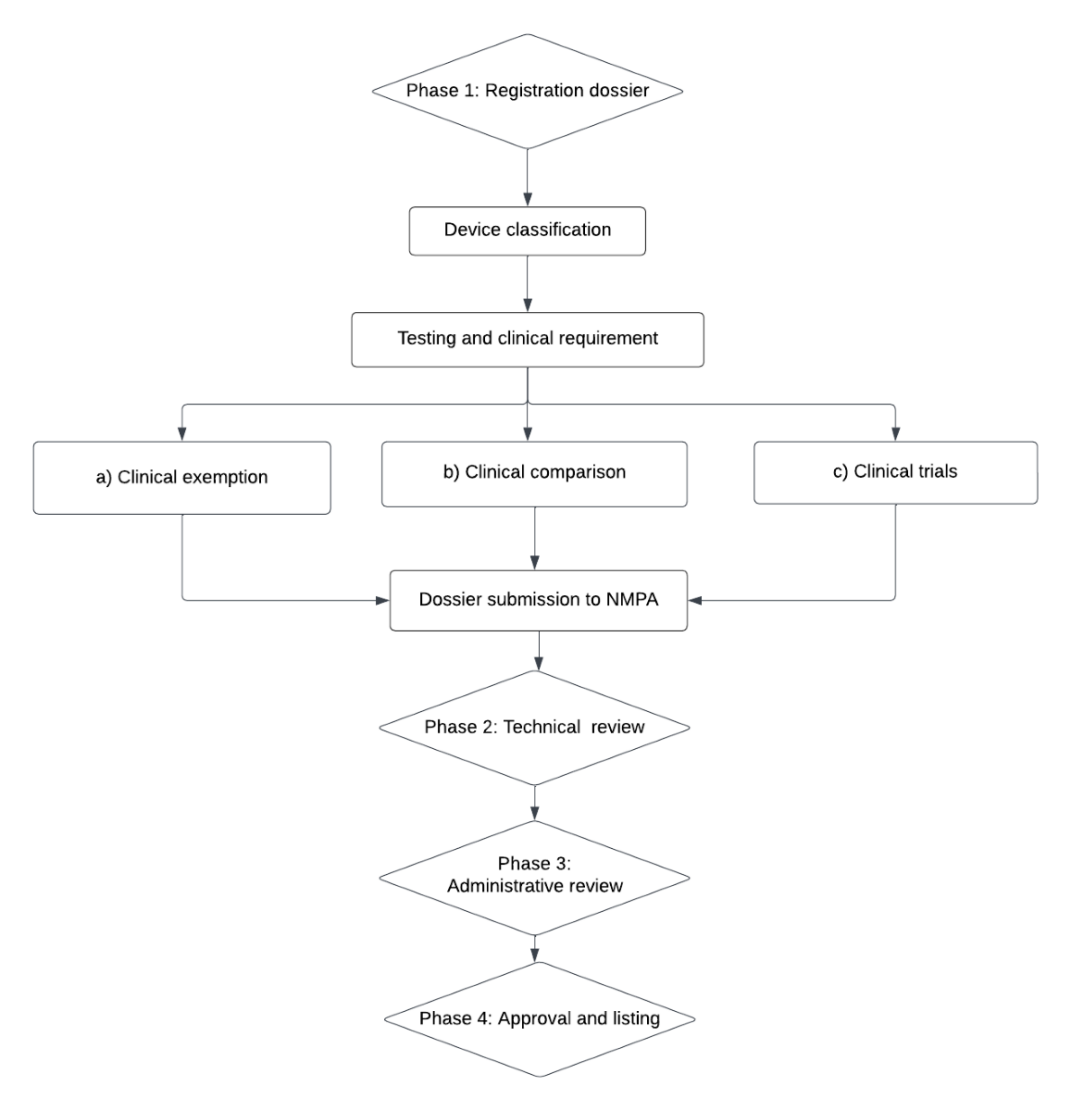
Medical device software registration process. NMPA: National Medical Products Administration.

Ordinarily, medical software devices, regardless of whether they use AI or not, are typically not categorized under class I. Within the context of classes II and III devices, the registration process typically takes around 18 months if no clinical trials are required. However, once clinical trials are needed, the registration timeline can extend to around 36 months or sometimes even longer. The exact timeline is dependent on the complexity of the device and the associated clinical data. It is of particular importance to note that certain devices may qualify for expedited processing through a fast-track pathway. Under these circumstances, not only can registration fees be exempted, but the registration timeline is accelerated, as it is typically condensed to approximately 50 working days. Currently, there are 2 software devices that have been designated under the Fast Track pathway in China, namely, an implantable left ventricular assist software system and a coronary CT fractional flow reserve calculation software, identified by license numbers 20213120987 and 20213210270, respectively. Refer to Figure 3 for an overview of the registration process for Class II and Class III software devices.

In accordance with the Notice of the Chinese NMPA, which relates to the issuance of 5 technical guidelines, including the Technical Guidelines for Clinical Evaluation of Medical Devices (number 73 of 2021), it is evident that there exist 3 distinct pathways for meeting the required clinical standards (see Figure 3). These pathways encompass (1) a clinical exemption, (2) a clinical comparison, and (3) a clinical trial, each associated with a gradient of clinical requirements ranging from low to high. Exemptions can be obtained if the device is part of the catalog of devices that are exempt. For medical devices not encompassed within the “Catalog of Medical Devices Exempt from Clinical Trials,” the pathway of conducting a comparative analysis with similar products already available on the market can be explored. This can be realized through the systematic collection and meticulous analysis of clinical data and other pertinent evidence, thereby proofing their equivalence and thus expediting the clinical evaluation process.

The need to conduct clinical trials for AI medical devices is thus not universally mandated. Furthermore, if clinical trials are required, then it is not determined solely by their classification. The requirement to run a clinical trial depends on the intent and the application. The NMPA's “Guidelines for the Evaluation of Artificial Intelligence Medical Devices,” states that for functionalities that do not entail decision-making assistance and are grounded in core operations, a rigorous comparative analysis with similar medical devices within the same category is required. However, for decision-assistive functions underpinned by core algorithms, a comparative analysis with equivalent medical devices within the same category is only advocated. Nonetheless, the devices selected for comparison should ideally have undergone comprehensive clinical trials, although historical data may be acceptable in certain circumstances. Finally, novel functions, algorithms, and applications should be subjected to exhaustive clinical trials to ensure their efficacy and safety within the clinical domain.

## Cultivating AI Software Devices: An Emerging Trend in China

Following the introduction of the new generation AI development plan, major shifts have occurred in both investment and policy domains to align with the overarching objectives of this plan. Notably, the NMPA, as China's regulatory authority for medical products, has promulgated a series of regulations in line with the thematic contours of the plan. In 2022, the NMPA initiated a digital health program. Over the course of 5 years, the NMPA, operating as a subsidiary of the Chinese government, has enacted a suite of regulations to govern the medical device industry, a selection of which is delineated in Table 1. These encompass pivotal documents such as the “Key Points of Deep Learning Decision-Making Assisting Medical Device Software Review [13]” and the “Guidelines for the Classification and Designation of Artificial Intelligence Medical Software [14],” as well as the “Guidelines for Medical Device Software Registration and Review [15]” and a duplicate mention of the “Guidelines for the Classification and Designation of Artificial Intelligence Medical Software” [16].

China's concerted efforts in this domain have manifested in substantial investments and the development of numerous medical software applications. An illustrative milestone occurred in the year 2020 when the first AI-based diagnostic software received approval in China, specifically for employment in CT image AI-assisted diagnostic software products. As of 2023, an exhaustive review of the NMPA website has revealed that China has granted approvals for more than 50 AI medical device products rooted in deep learning technology [26]. These products, predominantly classified as medical software, serve as pivotal aids in diagnostic processes encompassing CT images, fundus images, and magnetic resonance images, and are strategically deployed within specialized fields such as radiology, ophthalmology, and cardiology. Moreover, regional governments seem to have demonstrated proactive engagement with the evolving landscape.

## Challenges Posed by Software and AI in Medical Devices

As AI technology develops further, regulators will also face the challenge of applying regulatory safeguards to these novel technologies. The technical complexity of certain medical software solutions warrants the description of these systems as a “black box,” due to their inherent opacity [27,28]. In addition, traditional frameworks for regulation are not suitable for adaptive AI and ML technologies, since the algorithms are constantly learning and making changes [29]. Therefore, digital health care solutions provide a different set of challenges to regulators and the traditional fixed regulatory framework is not suitable for this type of AI device. At present, governmental agencies in the United States, the European Union, and China have all issued new regulatory methods or frameworks for MDSW to help cope with the changing landscape.

The regulation of AI devices is to ensure safety, quality, and reliability requirements are met. One key concern is the ”unlocked“ nature of these devices. ”Locked“ devices mean that the algorithm provides the exact same result for a (specific) given input [29]. This contrasts with an “unlocked” algorithm, which represents a continuous learning algorithm. The “unlocked” algorithm is also known as an adaptive algorithm, and it changes its behavior using a predefined learning process that provides time-based updates from new data with the overall aim of improving its clinical performance. This algorithm continuously changes the input-output relationship. Thus, for a given set of inputs, the output may be different before and after these changes are implemented. This means that after a “locked” device has been approved and given access to the market, the device can continue to self-learn and thus alter its performance in comparison to when it was first approved. In this situation, it is difficult for the clinicians or the authorities to fully trust the device before they use it in practice. So far, the Food and Drug Administration (FDA) has not yet approved a device that integrates continual learning AI, as they have only granted approval to locked systems [29].

The FDA has enacted the Digital Health Innovation Action Plan [30], with the aim of building a more dynamic approval process with precertification for companies that will then have the ability to change the characteristics of a product without needing ongoing FDA assessment. This enterprise-based approach (precertification program) is very different from traditional medical device regulation. The FDA adopted the precertification program together with the total product life cycle database to screen for eligible organizations. They also adopted a “predetermined change control plan.” This plan provides a complete approach based on the total product lifecycle in a way that manages the risk to patients in a controlled manner.

The European Union (EU) also enacted new directives to regulate this fast-changing technology domain. They include the general data protection regulation (GDPR), cybersecurity directive, medical devices regulation, and in vitro diagnostic medical device regulation. The GDPR and the Cybersecurity Directive took effect in May 2018, whilst the medical devices regulation was applied in May 2021, with the in vitro diagnostic medical device regulation following suit a year later. These recent changes further highlight the moving landscape of regulations on a global scale.

Besides the apprehension about the increase in regulatory complexity for AI and ML, other aspects are also starting to raise concerns. Among those are ethical considerations, cybersecurity, and the reproducibility of the performance. These aspects are briefly discussed below.

## Ethical Considerations

Ethical issues have been intensely debated since the start of AI technology development. In the medical field, obvious questions are posed with regard to data privacy, physician dependency, and potential bias in post-GDPR algorithms, as well as concerns about changes in the doctor-patient relationship [31]. People are also concerned about algorithmic fairness and potential biases. The algorithms are data-driven, and it could be that the data used might not meet the required ethical standards.

In April 2019, the National Artificial Intelligence Standardization General Group in China issued the “Artificial Intelligence Ethical Risk Analysis Report” [32], which further clarified that the principle of fundamental human interests should be considered from three viewpoints: (1) the impact on society, (2) the AI algorithm, and (3) the used data. All these ethical concerns need to be navigated in order to create appropriate technology that can be used in the clinic.

## Reproducibility

Reproducibility is also an important item in the field of AI. Nowadays, many AI devices face a problem as their outcomes are not verifiable by third parties [33]. The reasons for this can be related to the quality of the data, data inputs, the transparency of data, or the code used for processing, to name a few factors [34]. There is a particular concern for adaptive AI, as the data upon which the model would be built changes, which in turn can trigger a change in outputs. Consideration should also be given to the need for detailed information on the data processing and training pipelines, as this is often lacking [35].

NMPA issued a document (number 8, 2022) [16] that requires reproducibility evidence from the sponsor in multiple dossier sections. These sections include user need analysis, algorithm property evaluation, and algorithm verification and validation. In the algorithm property evaluation, it suggests that applicants should consider requirements such as false negatives and false positives (indicators and relationships), repeatability, reproducibility, and robustness. At the same time, all factors that affect algorithm performance should be analyzed, and their degree of influence should be determined. This includes things such as the acquisition equipment, acquisition parameters, disease composition, and lesion characteristics, among others. Taking these into account will improve algorithm interpretability and it can serve as the basis for software verification and validation [36].

## Cybersecurity

Like other computer systems, MDSW can be vulnerable to security breaches [37]. It has been suggested that 53% of connected medical devices contain critical vulnerabilities, and health care professionals struggle to maintain the inventories of connected devices [38]. For many years, cyberattacks have been identified as the top health tech hazard within this space [38]. The FDA indicates that cybersecurity issues could directly impact the safety and effectiveness of the device, as further harm can be caused to the patients who are using them [37]. Reducing cybersecurity risks is especially challenging while medical devices interact with human bodies; as a result, it becomes a multidisciplinary problem concerning engineering, computer science, medical, and physical sciences.

The IMDRF issued Principles and Practices for Medical Device Cybersecurity in 2020 [39], which introduces a “total product life cycle” risk reduction plan for cybersecurity. Authorities are now focusing on scrutinizing applicants’ dossiers to make sure a thorough plan has been designed, which contains a risk management process, risk analysis, risk control or residual risk, post-marketing plan, etc. In 2022, the NMPA released a new version of principles of medical device cybersecurity technical evaluation [40], which also ensures data confidentiality, integrity, availability, authenticity, accountability, nonrepudiation, and reliability are covered according to GB/*t* 29246-2012. The NMPA suggests that applicants make sure that the risk management method is applied throughout the whole life cycle to ensure patient safety. They will focus on quality control across all stages mentioned before in both the pre- and postmarket phases.

## Future Directions

Since China joined the IMDRF in 2013 [41], China has adopted and referenced international regulatory methods when formulating its own regulations. Regulatory similarities between China and other countries have been witnessed and demonstrated. However, China also has its own local requirements, standards, and regulatory ideologies, which can be an additional layer of complexity for global manufacturers who want to bring their medical devices to the Chinese market.

There are different aspects for global manufacturers to pay attention to when they want to leverage US or EU experience for the Chinese market. In China, the focus is more on the maturity of the algorithm, which is different from the FDA sponsor qualification program. Differences in sample populations upon which the algorithm is built are another key consideration, in addition to the requirement to ensure data confidentiality and the protection of patients in a specific region. In the Regulatory Science Action Plan issued by NMPA in 2019 [42], there is a clear focus on AI, which suggests more regulations might be developed with an increased level of harmonization with the US, EU, or other markets. Nonetheless, regulatory inconsistency still exists between countries. The same device can be regulated very differently across borders, which poses global manufacturers with big challenges. Large, well-founded medical device companies usually have global regulatory affairs professionals that deal with this situation, but innovation may also arise from small research teams at universities or innovative small and medium enterprises. In this situation, the complexity of the regulatory environment will hinder the potential of influential new products to enter the market. The regulatory strategy will need to differ from region to region to provide the best possible match for each.

For example, in the United States, a high-tech device could be registered as a class II device if it is like a predicate device that has already been registered. In this case, the characteristics need to be the same, and there should not be any cause for concern with regard to the safety and effectiveness of the device. However, in China, manufacturers will need to refer to the classification catalog, which aims to classify the device based on its own safety and effectiveness. If it is a high-tech device, then it becomes more likely that it will be seen as a class III device in China. This means that the device will face more stringent registration requirements, including clinical evaluation and even trials. Manufacturers need to consider this when they start to map their market potential globally, as it could become a regulatory barrier for them.

Strategically, some manufacturers would choose to register their devices first in the United States and then explore China or other markets. The United States regulation is also focused on the sponsor criteria and “Current Good Manufacturing Practice” alongside the assessment of the software algorithm itself, which makes it more organization-centric [43]. Another registration strategy could be to register half of the medical devices that are in the development stage (also called “pipeline products”) in the United States and the other half in China. After getting feedback from both authorities, they can switch them over. In the United States, applicants of new devices can go through the De Novo premarket pathway or Breakthrough Device designation to register their technology [43]. In China, there exists a “Green Channel” for software with urgent medical needs.

It is imperative for international manufacturers and regulatory authorities to engage in collaborative endeavors aimed at delineating optimal regulatory pathways for each AI and ML product. Establishing a conducive environment where stakeholders can engage in reciprocal learning is of paramount importance. Enhanced comprehension of regional regulatory variations serves as a catalyst for fostering an environment conducive to mutual learning and collaboration. Bolstering global regulatory awareness in the health care technology sphere has the potential to catalyze new opportunities, ultimately yielding enhanced benefits for patients in the long term.

## Appendix

Multimedia Appendix 1 Medical Device Software Information in NMPA Translated from Medical Device Classification Catalog. NMPA: National Medical Products Administration.

## Abbreviations

AI: artificial intelligence
CT: computed tomography
EU: European Union
FDA: Food and Drug Administration
GDPR: general data protection regulation
IMDRF: International Medical Device Regulators Forum
MDSW: medical device software
ML: machine learning
NMPA: National Medical Products Administration
SaMD: software as medical device
SiMD: software in a medical device

## References

[1] Wang X, Peng Y, Lu L, Lu Z, Bagheri M, Summers R (2017). ChestX-Ray8: hospital-scale chest X-ray database and benchmarks on weakly-supervised classification and localization of common thorax diseases.

[2] Patel N, Michelini V, Snell J, Balu S, Hoyle AP, Parker JS, Hayward MC, Eberhard DA, Salazar AH, McNeillie P, Xu J, Huettner CS, Koyama T, Utro F, Rhrissorrakrai K, Norel R, Bilal E, Royyuru A, Parida L, Earp HS, Grilley-Olson JE, Hayes DN, Harvey SJ, Sharpless NE, Kim WY (2018). Enhancing next-generation sequencing-guided cancer care through cognitive computing. Oncologist.

[3] Ting DSW, Pasquale LR, Peng L, Campbell JP, Lee AY, Raman R, Tan GSW, Schmetterer L, Keane PA, Wong TY (2019). Artificial intelligence and deep learning in ophthalmology. Br J Ophthalmol.

[4] Szolovits P (2019). Artificial Intelligence and Medicine. Routledge.

[5] Forlenza GP (2019). Use of artificial intelligence to improve diabetes outcomes in patients using multiple daily injections therapy. Diabetes Technol Ther.

[6] Tzelios C, Nathavitharana RR (2021). Can AI technologies close the diagnostic gap in tuberculosis?. Lancet Digit Health.

[7] Notice of the state council on the new generation artificial intelligence plan. State Council of the People's Republic of China.

[8] Morrison WM (2013). China's Economic Rise: History, Trends, Challenges, and Implications for the United States.

[9] China Population in 2022. Worldometer.

[10] (2021). Chinese medical device industry: how to thrive in an increasingly competitive market?. Deloitte.

[11] Ceross A, Bergmann J (2021). Evaluating the presence of software-as-a-medical-device in the Australian therapeutic goods register. Prosthesis.

[12] Announcement of the NMPA on issuing the guiding principles for technical review of medical device software registration. NMPA.

[13] Key points of deep learning decision-assisting medical device software review. Artificial Intelligence Medical Device Innovation and Cooperation Platform.

[14] Announcement of NMPA on issuing the guiding principles for the classification and definition of artificial intelligence medical software products products (no. 47 of 2021). NMPA.

[15] Announcement of the center for device evaluation of the state food and drug administration on the release of the guidelines for the registration and review of medical device software (2022 revision) (2022 no. 9). CCFDIE.

[16] Notice of the center for device evaluation of the NMPA on issuing the guiding principles for the registration review of artificial intelligence medical devices (no. 8, 2022). CIRS.

[17] Software as a medical device (SaMD)). FDA.

[18] Announcement of the general administration on matters concerning the implementation of the catalogue of medical devices. NMPA.

[19] Soliman E, Mogefors D, Bergmann JHM (2020). Problem-driven innovation models for emerging technologies. Health Technol.

[20] Maci J, Marešová P (2022). Critical factors and economic methods for regulatory impact assessment in the medical device industry. Risk Manag Healthc Policy.

[21] Arnould A, Hendricusdottir R, Bergmann J (2021). The complexity of medical device regulations has increased, as assessed through data-driven techniques. Prosthesis.

[22] Regulations on the supervision and administration of medical devices. NPMA.

[23] (2020). YY/*t* 0664-2008 medical device software life cycle process. NMPA.

[24] Shankui R, Xiao J, Jian F, Chunqing Z, Xinhua Y (2019). Research on classification management of computer aided diagnosis software products. Chin J Med Devices.

[25] IMDRF (2014). Software as a Medical Device: Possible Framework for Risk Categorization and Corresponding Considerations. Published online.

[26] National medical products administration database. CDE.

[27] Pashkov V, Harkusha A (2016). Certain aspects on medical devices software law regulation. Wiad Lek.

[28] Pashkov VM, Harkusha AO, Harkusha YO (2020). Artificial intelligence in medical practice: regulative issues and perspectives. Wiad Lek.

[29] Proposed regulatory framework for modifications to artificial intelligence/machine learning (AI/ML)-based software as a medical device(SaMD)). FDA.

[30] Digital health innovation action plan. FDA.

[31] Dalton-Brown Sally (2020). The ethics of medical AI and the physician-patient relationship. Camb Q Healthc Ethics.

[32] Artificial intelligence ethical risk analysis report. Institute ETS.

[33] A call for greater transparency, reproducibility in use of artificial intelligence in medicine. Harvard THC.

[34] Cruz M, Kurapati S, Turkyilmaz-van DVY (2018). Software reproducibility: how to put it into practice?. OSFPREPRINTS.

[35] Haibe-Kains B, Adam GA, Hosny A, Khodakarami F, Waldron L, Wang B, McIntosh C, Goldenberg A, Kundaje A, Greene CS, Broderick T, Hoffman MM, Leek JT, Korthauer K, Huber W, Brazma A, Pineau J, Tibshirani R, Hastie T, Ioannidis JPA, Quackenbush J, Aerts HJWL, Massive Analysis Quality Control (MAQC) Society Board of Directors (2020). Transparency and reproducibility in artificial intelligence. Nature.

[36] Key points of deep learning decision-assisting medical device software review. Artificial Intelligence Medical Device Innovation and Cooperation Platform.

[37] Cybersecurity. FDA.

[38] McKeon J Cyberattacks will be the top health tech hazard this year, ECRI says. HEALTH IT SECURITY.

[39] Group MDCW (2020). Principles and practices for medical device cybersecurity. IMDRF.

[40] Neishen A Principles of medical device cybersecurity technical evaluation. IMDRF.

[41] Yue M, Wenwen Z, Shuo P, Yiwu H, Bin L, Zhong L (2019). IMDRF interpretation of personalized medical device terms. China Pharm Affairs.

[42] NMPA launched the china drug regulatory science action plan. NMPA.

[43] He J, Baxter SL, Xu J, Xu J, Zhou X, Zhang K (2019). The practical implementation of artificial intelligence technologies in medicine. Nat Med.

