# Supplementary material for: Regulatory Frameworks for AI-Enabled Medical Device Software in China: Comparative Analysis and Review of Implications for Global Manufacturer
Source: JMIR AI. 2024 Jul 29;3:e46871. doi: 10.2196/46871 (PMC11319888; doi:10.2196/46871)
Supplement: Multimedia Appendix 1 [file ai_v3i1e46871_app1.pdf]

Appendix 1. Medical Device Software Information in NMPA Translated from *Medical Device Classification Catalog*

No.	Primary product category	Secondary product category	Product Description	Intended Use	Example of product name	Classification
01	Treatment Planning Software	01 Radiation therapy planning system software	It usually consists of a software installation CD (or downloading the installation program from the Internet). Usually (not required), one or more specific algorithms are used to estimate the absorbed dose distribution of human organs.	Used to formulate the patient's radiation treatment plan.	Radiation treatment planning system software, gamma-ray stereotactic radiation treatment planning system software, radioactive seed source implantation treatment planning system software	III
		02 Radiation Therapy Assistant Software	It usually consists of a software installation CD (or downloading an installation program from the Internet). Provide, define, or display the setting data of the treatment machine; manually input data or directly import data from other equipment; record the data of the entire treatment stage.	It is used to compare the current parameters and preset parameters of the radiotherapy machine before the start of the planned radiotherapy and before the start of each treatment phase, and to record the data of the actual treatment phase.	Radiation therapy recording and verification system software	III
			It usually consists of a software installation CD (or downloading an installation program from the Internet). Before radiotherapy, use the acquired image information and analysis and processing results to determine the target coordinates or position.	Used to assist in the completion of radiotherapy.	Radiation therapy contour drawing software, radiation therapy simulation positioning software	III

No.	Primary product category	Secondary product category	Product Description	Intended Use	Example of product name	Classification
		03 Surgical planning software	It usually consists of a software installation CD (or downloading an installation program from the Internet). Use the obtained image information and the results of its analysis and processing to formulate a surgical plan.	Used to make a surgical plan before surgery. Except for dentistry and ear nose and throat.	Stereotactic surgery planning software, surgery simulation software, surgery planning software	III
			It usually consists of a software installation CD (or downloading an installation program from the Internet). Use the acquired image information and its analysis and processing results to formulate dental and ENT surgery plans or programs.	It is used to make surgical plans before dentistry and ENT surgery.	Digital implant design software, dental restoration design software	II
02	Image processing software	01 Medical image storage and transmission system software	It usually consists of a software installation CD (or downloading an installation program from the Internet). The image and/or video signal output by the medical imaging equipment is collected and saved to the computer hard disk for the processing of image reception, transmission, display, storage, and output between various departments of the medical department and/or between hospitals.	Used for medical image receiving, transmission, display, storage, output and other processing, for clinical diagnosis and treatment.	Image archiving and transmission system software, medical image management and communication system software, medical image archiving and communication system software	II

No.	Primary product category	Secondary product category	Product Description	Intended Use	Example of product name	Classification
		02Medical image processing software	It usually consists of a software installation CD (or downloading the installation program from the Internet). Use image processing methods to perform three-dimensional reconstruction and registration of medical images.	Used to process medical images from single mode or multiple modes.	Ultrasound image management software, endoscopy graphics workstation software, digital ultrasound workstation software, magnetic resonance image processing software, nuclear medicine workstation software, CT image processing software, X-ray angiography image processing software, digital X-ray image processing software	II
03	Data Processing Software	01 The monitoring software	It usually consists of a software installation CD (or downloading the installation program from the Internet). Obtain data from the monitoring equipment through data communication, and display and alarm in real time.	Used to obtain data from monitoring equipment, centralized real-time display, and alarm.	Central monitoring workstation software, central monitoring management software, central monitoring information center software, pregnancy-induced hypertension syndrome monitoring software	II
		02 Physiological signal processing software	It usually consists of a software installation CD (or downloading the installation program from the Internet). Analyze, process and/or transmit the collected physiological signals such as brain electricity, electrocardiogram, and myoelectricity.	It is used to analyze, process and/or transmit physiological signals such as brain electricity, electrocardiogram, and myoelectricity.	Holter analysis software, ECG workstation software, ECG data management software	II

No.	Primary product category	Secondary product category	Product Description	Intended Use	Example of product name	Classification
04	Decision Support Software	01 Drug calculation software	It is usually composed of software installation CD (or download the installation program from the Internet). Calculate the drug injection plan based on the pharmacokinetics and/or drug model, the patient's physiological parameters and physical signs, and provide recommendations for clinical injection of drugs.	Used to provide recommendations for clinical injection of drugs.	Insulin injection calculation software	III
		02 Computer Aided Diagnosis/Analysis Software	It usually consists of a software installation CD (or downloading an installation program from the Internet). Using image processing and/or data processing technology, computer software automatically recognizes the lesion, and provides clinical diagnosis and treatment basis and/or recommendations for the nature of the lesion.	The computer software automatically recognizes the lesions and provides clinical diagnosis and treatment basis and or suggestions for the nature of the lesions.	Mammary X-ray imaging computer-aided diagnosis software, colon computer-aided diagnosis software, lung computer-aided diagnosis software, breast ultrasound-assisted diagnosis software	III
			It usually consists of a software installation CD (or downloading an installation program from the Internet). Analyze images or data and give clinical reference values.	Analyze images or data and give clinical reference values.	Bone Density Computer Aided Testing Software	II
		03 Chinese medicine diagnosis and treatment software/	It usually consists of a software installation CD (or downloading an installation program from the Internet). Use the relevant theories of TCM syndromes and treatments and use data statistics and other methods to realize the analysis and diagnosis of various symptoms and/or provide treatment suggestions.	It is used to realize the analysis and diagnosis of various symptoms and/or provide treatment suggestions.	Auxiliary diagnosis and treatment software, Chinese medicine diagnosis and treatment software	II

No.	Primary product category	Secondary product category	Product Description	Intended Use	Example of product name	Classification
05	In Vitro Diagnostic Software	01 Medical microscopy image analysis software	It usually consists of a software installation CD (or downloading an installation program from the Internet). It has the functions of acquiring, transmitting, synthesizing, observing, analyzing, processing and reporting the images obtained from various microscopic equipment.	It is used for auxiliary diagnosis, analysis and archiving of images obtained from various microscopic equipment.	Medical pathology image acquisition software, medical microscopic image analysis software, chromosome analysis software, urine sediment analysis software	II
		02 Screening and analysis software	It usually consists of a software installation CD (or downloading the installation program from the Internet). Through the analysis and calculation of clinical, biochemical, immune and other measurement data, the diagnosis and evaluation of diseases can be carried out.	It is used to screen and evaluate diseases.	Prenatal screening and analysis software, Down syndrome prenatal screening and analysis software, neural tube malformation prenatal screening and analysis software, 21 trisomy syndrome risk calculation software, 18 trisomy syndrome risk calculation software, neural tube defect risk Calculation software, blood glucose data analysis software	II
06	other software	01 Rehabilitation training software	It usually consists of a software installation CD (or downloading the installation program from the Internet). It is a software system composed of hierarchical inspection, training and auxiliary treatment modules, or a single module.	Used for auxiliary treatment and rehabilitation training.	Visual function training software for children with amblyopia, visual function inspection training software	II
